# An Interesting Case

**Published:** 1895-02

**Authors:** Louis Olry Lusson

**Affiliations:** Ardmore, Pa.


BY LOUIS OLRY LUSSON, ARDMORE, PA.

### AN INTERESTING CASE.

BY LOUIS OLRY LUSSON, ARDMORE, PA.

ON June 22, 1893, I was called by a Mr. G. to see a yearling filly by the famous hackney stallion "Matchless," of Londresboro. The filly was in a pasture lot with a number of other colts, and had been noticed by one of the men an hour before to be apparently all right. When I arrived I found her in one corner of the paddock unable to rise; and, after calling some assistance, endeavored to place her on her feet. I found, however, that her hind quarters were incapable of supporting her weight. She was placed on a sled and removed to a box stall and placed in slings. Improvement was so slow that she was kept in the slings for eight or nine weeks. She was then allowed the freedom of the box stall. She could stand and walk (with a wobble), but if she attempted to turn suddenly around would fall. She was placed in a paddock by herself, and could trot and gallop, but still walked with a wobble, and in trotting would " grab " her forequarters, and in attempting to stop or turn suddenly would fall.

My diagnosis was an impingement of the cord, caused by a fracture of the body of the vertebra in falling, as when found the filly was in such a position that to me it appeared as though the colts might have been all running toward the corner, suddenly stopped, and in so doing might have knocked down this filly and caused the injury. The history, symptoms, and partial recovery assimilate very closely a case of infantile paralysis.

I made it my duty to see this filly now and again for about a year, and at several different times gave a strong alterative and nerve tonic, to try to repair what I thought an inactivity of the cord. During this period the animal gradually improved. She would still sway behind when trotting slowly or walking, but would not fall. My friend, Dr. —, has been treating the above gentleman's stock for the past year, and he informs me that the " Matchless " filly still has the same unsteady gait and had developed a beautiful case of osteoporosis of the superior and inferior maxilla. The problem was solved and my diagnosis correct—a fracture of the vertebra, sustained by a fall or concussion, assisted by the change in the bony structure, had caused pressure on the spinal cord and consequent partial paralysis.

---

THE results of a complete census of farm animals, conducted by the *Orange Judd Farmer*, and covering every county in the country, appears in the January issue. The decline in values during the past year is very heavy, the aggregate value of all classes being $1,922,600,000, against $2,262,400,000 a year ago, a shrinkage of 15 per cent. The total number of horses on farms is given at 16,082,000, valued at $678,807,000, a decline of $115,000,000 in value; mules, 2,341,000, valued at $107,574,000; cows, 17,451,000, value $387,576,000; cattle, 32,398,000, value $458,303,000; sheep, 35,819,000, value $60,758,000; hogs, 47,061,000, value $229,568,000.

